# The Spiritual Dimensions of Caring for a Family Member With Early-Stage Dementia

**DOI:** 10.1093/geroni/igaa057.1142

**Published:** 2020-12-16

**Authors:** Jocelyn McGee, Dennis Myers, Rebecca Meraz, Janet Galarza, Morgan Davie, Jensen Smith

**Affiliations:** Baylor University, Waco, Texas, United States

## Abstract

There are approximately 16.1 million family members caring for persons with Alzheimer’s Disease and other dementias in the USA. However, few studies have examined the role of spirituality/religiosity as it relates to the caregiving experience, particularly in family members of persons in the early-stages of dementia. In this cross-sectional qualitative study, one-on-one, in-depth, 60-90 minute interviews were conducted with family members of persons assessed to be in the early-stages of dementia (n = 26). A structured interview, “The Spiritual and Religious Dimensions of Living with Dementia” developed by McGee, J. & Carlson-Zhao, H. (2012), was utilized. Interviews were recorded and transcribed by members of an interdisciplinary team of gerontology researchers (nursing, psychology, and social work). Narrative data were examined through thematic analysis approach, supported by NVivo (version 12.0) software, and a modified constant comparison analysis approach (Glaser & Strauss, 1967). Participants reported a diverse range of spiritual/religious beliefs, practices, and experiences including how these intersected with their adaptation to the opportunities and challenges of the caregiver role. The importance of intrapersonal processing, family connection, and community support for managing the ambiguity of this role was expressed. The interview questions were reportedly therapeutic for some participants suggesting the need to include similar questions as part of the assessment in clinical care settings. Indeed, interviewing caregivers of people with early-stage dementia may serve to improve clinical outcomes by identifying important aspects of spiritual/religious coping that can be encouraged as well as spiritual struggles that need to be addressed. Additional research is needed.

